# Antioxidant Activity and Phytochemical Characterization of *Senecio clivicolus* Wedd.

**DOI:** 10.3390/molecules23102497

**Published:** 2018-09-29

**Authors:** Immacolata Faraone, Dilip K. Rai, Lucia Chiummiento, Eloy Fernandez, Alka Choudhary, Flavio Prinzo, Luigi Milella

**Affiliations:** 1 Department of Science, University of Basilicata, 85100 Potenza, Italy; lucia.chiummiento@unibas.it (L.C.); prinzo92@live.it (F.P.); 2 Teagasc Food Research Centre, Ashtown, Dublin D15KN3K, Ireland; Dilip.Rai@teagasc.ie (D.K.R.); alkachoudhary12@gmail.com (A.C.); 3 Faculty of Tropical AgriSciences, Czech University of Life Sciences Prague, Kamýcká 129, 16500 Prague 6, Suchdol, Czech Republic; eloy@ftz.czu.cz

**Keywords:** Asteraceae, *Senecio clivicolus*, DPPH, *beta*-carotene bleaching, RACI, phenolic characterization, UHPLC-MS/MS, polyphenols, flavonoids, health-promoting compounds

## Abstract

Antioxidant phytochemicals play a key role in oxidative stress control and in the prevention of related disorders, such as premature aging, degenerative diseases, diabetes, and cancer. The aim of this study was to investigate the potential antioxidant activity and the phytochemical profile of *Senecio clivicolus* Wedd., a perennial shrub, belonging to the Asteraceae family. Despite the wide interest of this family, this specie has not been investigated yet. *S. clivicolus* aerial parts were extracted with 96% ethanol. Then, the ethanol extract was fractionated by liquid/liquid extraction using an increasing solvents polarity. Total polyphenol and terpenoid contents were measured. Moreover, the antioxidant activity was evaluated by six different complementary in vitro assays. The Relative Antioxidant Capacity Index (RACI) was used to compare data obtained by different tests. The sample showing the highest RACI was subjected to characterization and quantitation of its phenolic composition using LC-MS/MS analysis. The ethyl acetate fraction, investigated by LC-MS/MS analysis, showed 30 compounds, most of them are chlorogenic acid and flavonoid derivatives. To the best of our knowledge, this is the first report about the evaluation of antioxidant activity and phytochemical profile of *S. clivicolus*, underlying the importance of this species as a source of health-promoting phytochemicals.

## 1. Introduction

Plants are an important source of bioactive compounds with antioxidant capacity and during the last years, the interest for the use of natural products has significantly increased. The protective effects of plant secondary metabolites can be attributed to direct scavenging activities against reactive oxygen species (ROS), as well as to the induction of intracellular antioxidant effect [1,2]. In fact, different epidemiological studies have shown as the decrease of premature death and mortality from cancer or other chronic diseases are associated with antioxidant-rich diet including fruit, vegetables, and other botanicals [3,4]. Reactive oxygen and nitrogen species (RNS) are physiologically produced during metabolic processes and especially during electron transport chain reactions [4], low concentration of these species is essential for several biochemical processes. An important endogenous antioxidant system compensates the production of ROS, RNS, and other free radicals, but an overall increase in cellular levels of these unstable molecules above the cells defenses results in oxidative stress that can ultimately cause cell death [5]. Oxidative stress is responsible for toxicity and damage of cell components, including nucleic acids, proteins, and lipids [3,6] and it has been recognized responsible for the onset of several diseases, including liver diseases, diabetes, neurodegenerative, and cardiovascular diseases. Thus, dietary antioxidants have been proposed as therapeutic agents to counteract oxidative stress-based diseases [4]. 

*Senecio* is one of the largest genus of the Asteraceae family, with a wide distribution in the world, consisting more than 1500 species of herbs, shrubs, vines, and trees. Many of these species grow in Bolivia, a central country of South America, with an extremely rich biodiversity of endemic species [7]. The traditional use of medicinal plants by various indigenous populations in Bolivia has been documented in the literature [8,9,10,11,12,13]. In particular, traditional medicine used extracts of leaves and roots of several *Senecio* species as a remedy for fever, cough, stomach pain, gastric ulcer, for the treatment of diabetes, skin wounds, and as a vasodilator, antiemetic, and anti-inflammatory agents [14].

*Senecio clivicolus* Wedd. grows in the mountain region of western Bolivia, where it is known as “*chiñi waycha*” or “*waycha negra*”. The natives used this perennial shrub as a remedy for stomach pain and diarrhea [14,15]. Moreover, this plant extract was used against fungal infections in the skin [16]. 

Pyrrolizidine alkaloids, eremophilanes, terpenes, and furanoeremophilanes were reported in previous phytochemical studies on *S. clivicolus* and other species belonging to *Senecio* genus [17,18,19,20]. These metabolites have several important properties as insect antifeedant, antifungal, cytotoxic, antioxidant, anti-inflammatory, and antimicrobial agents. Some furanoeremophilanes, such as cacalone compound, have a radical scavenging and antioxidant activity [14,21]. 

Following our investigation of medicinal plants belonging to the Asteraceae family [22,23], and due to the ethno medical use of this specie, the present study was focused on the investigation of total polyphenolic content (TPC), the potential antioxidant activity, the structural characterization and quantification of secondary metabolites present *S. clivicolus* aerial parts.

Extracts were analyzed, using six different complementary assays, for their radical-scavenging activity against 2,2-diphenyl-1-picrylhydrazyl (DPPH), 2,2′-azino-bis-(3-ethylbenzothiazoline-6-sulfonic acid) (ABTS), superoxide anion (O_2_^−^) and nitric oxide (NO) radicals, for their ferric reducing antioxidant power (FRAP) and the capacity to inhibit lipid peroxidation by *β*-carotene bleaching test (BCB). Then, the phytochemical profile was performed by LC-ESI-MS/MS analysis and the identification and quantification of polyphenols were achieved using commercially available standards [24]. 

To the best of our knowledge, this is the first report about the evaluation of antioxidant activity and phytochemical profile of *S. clivicolus*, underling the importance of this specie as a source of health-promoting phytochemicals.

## 2. Results and Discussion

### 2.1. The Extraction Yield and the Influence of Solvents on Total Polyphenolic and Terpenoid Content

The dried aerial parts of *S. clivicolus* were extracted by dynamic maceration using 96% ethanol [25]. The extraction yield showed a value of 27.06%, higher than other reports. In fact, it has been reported that the extraction yield of the aerial parts of other species of *Senecio* with 95% ethanol (yield of 5.60% in *S. biafrae* [26] and 12.57% in *S. aegyptius* [27], methanol (yield of 13.85% in *S. gibbosus* [28]) or water (yield of 13.60% in *S. candicans* [29]) was considerably lower than values obtained in this study. 

**Figure 1:**
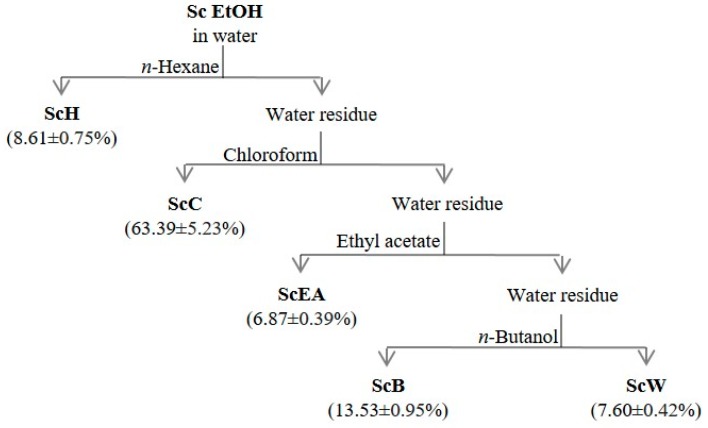
Extraction yields of *S. clivicolus* EtOH extract partitioned fractions. Results were expressed as mean ± standard deviation of the triplicate experiments. Samples are crude ethanol extract (Sc EtOH), *n*-hexane fraction (ScH), chloroform fraction (ScC), ethyl acetate fraction (ScEA), *n*-butanol fraction (ScB), and water fraction (ScW).

Then, liquid/liquid partition of a part of the crude ethanol extract (20.00 g) was performed for three times using increasing polarity solvents (*n*-hexane, chloroform, ethyl acetate and *n*-butanol); thus, a separation of the compounds was obtained based on the affinity with the solvent used [30,31]. The extraction yield of dry *n*-hexane (ScH), chloroform (ScC), ethyl acetate (ScEA), *n*-butanol (ScB) and water (ScW) fractions is reported in Figure 1. The chloroform fraction showed the highest extraction yield (63.39 ± 5.23%); instead, the water and ethyl acetate fractions demonstrated the lower extraction yields, 7.60 ± 0.42 and 6.87 ± 0.39%, respectively.

**Figure 2:**
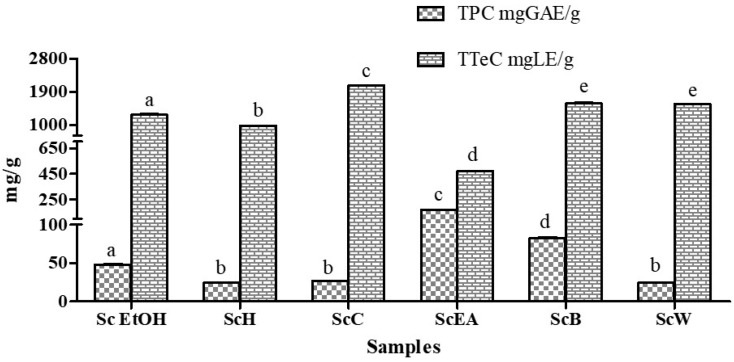
Total polyphenolic content (TPC) and total terpenoids content (TTeC). Results were expressed as mean ± standard deviation in mg of gallic acid equivalents per gram of dried sample (mg GAE/g), and in mg of linalool equivalents per gram of dried sample (mg LE/g). In each tests, the values with the same letter are not significant different at the *p* < 0.05 level, according to one-way analysis of variance (ANOVA). Samples are crude ethanol extract (Sc EtOH), *n*-hexane fraction (ScH), chloroform fraction (ScC), ethyl acetate fraction (ScEA), *n*-butanol fraction (ScB), and water fraction (ScW).

Total phenolic content (TPC) of Sc EtOH and its fractions was carried out based on the reaction of the samples with Folin-Ciocalteu reagent, which results in a blue colored solution whose intensity was directly proportional to the amount of phenolic compounds present. Gallic acid was used as standard and results were expressed as milligram of gallic acid equivalents per gram of dry extract (mg GAE/g) and shown in Figure 2. TPC ranged from 24.32 ± 0.43 to 170.11 ± 1.49 mg GAE/g in ScW and ScEA fractions, respectively. Out of all samples, ScEA had the highest value, followed by ScB and Sc EtOH (82.50 ± 1.94 and 48.31 ± 1.32 mg GAE/g, respectively). 

Moreover, total terpenoids content (TTeC) was evaluated for their characteristic reddish brown color at 538 nm. The linalool, a monoterpene, was used as standard reagent and results (Figure 2) were expressed as mg of linalool equivalents per gram of dry sample (mg LE/g). TTeC ranged from 470.77 ± 3.61 to 2074.24 ± 12.76 mg LE/g in ScEA and ScC fractions, respectively. ScC, followed by ScB, ScW, and Sc EtOH, exhibited higher values.

Results demonstrated as the yield of phenolic and terpenoid clearly differs on the basis of solvent polarity. Fractions obtained by using more polar solvents reported the highest total polyphenols, indicating that the majority of polyphenolic compounds in the aerial parts of *S. clivicolus* could be of polar nature, data congruent with what reported previously in literature [32].

### 2.2. Antioxidant Activity

Low concentrations of biological radicals are important in the human body; in fact, they are involved in biological functions as vascular homeostasis, neurotransmission, antimicrobial, antioxidant and antitumor activities. In contrast, the overproduction of biological radicals is associated with several diseases [1]. Phenolic compounds are gaining attention due to their significant antioxidant activities. Different in vitro assays were reported for the measurement of antioxidant activity on foods and plants and it has been demonstrated as more than one method is necessary to elucidate the antioxidant capacity of samples because these assays differ in the principles and experimental conditions. In this study, the antioxidant activity of the Sc EtOH extract and its fractions (ScH, ScC, ScEA, ScB, and ScW) were tested using six different complementary in vitro antioxidant assays. 

In particular, the radical scavenging activity was evaluated by using synthetic cationic and neutral (ABTS and DPPH) and physiological (superoxide anion and nitric oxide) radicals. The ScEA showed the highest radical scavenging-activity in all investigated tests (Table 1). In particular, the ScEA reported 409.53 ± 9.53 mg TE/g and 317.53 ± 5.81 mg TE/g values in ABTS and DPPH assays respectively, followed by ScB and Sc EtOH. Instead, in both assays, the lowest activity was found in ScH and ScC fractions.

The ability of samples to scavenge superoxide anion and nitric oxide was expressed as IC_25_, as it was not possible to reach 50% inhibition at tested concentrations, and the results were compared with ascorbic acid. The *S. clivicolus* extract and fractions caused a dose-dependent inhibition in the superoxide anion assay (SO) and ScEA fraction showed the highest inhibition, that means the lowest IC_25_ value (IC_25_ of 0.08 ± 0.00 mg/mL), followed by ScC, ScH, and ScB (Table 1). These samples showed a higher activity than that measured for ascorbic acid (IC_25_ of 0.26 ± 0.02 mg/mL).

The scavenging ability against the biological nitric oxide (^·^NO) was not detectable in any fraction, but ScEA showed a dose-dependent inhibition ability (IC_25_ of 1.11 ± 0.05 mg/mL); its value was four times lower than ascorbic acid (IC_25_ 4.78 ± 0.09 mg/mL), demonstrating its effectiveness.

The reducing ability of samples in a redox reaction was assessed using the FRAP test. ScEA fraction also in this case showed the highest activity (507.66 ± 5.26 mg TE/g) followed by ScB fraction (184.18 ± 4.59 mg TE/g). ScC and ScH were again the less active (Table 1).

To get a broader overview of the antioxidant potential of the plant complex of *S. clivicolus* samples, the *β*-carotene bleaching test (BCB) was conducted. The phenolic compounds compete with *β*-carotene for binding to the radical derived from linoleic acid and prevent the destruction of the conjugated system of the molecule responsible for coloring, for which, the higher is the antioxidant activity of the extract, the greater is the concentration of *β*-carotene in solution. Results were expressed as percentage of antioxidant activity (%AA) at the initial sample concentration of 1 mg/mL (Table 1). The analysis evidenced that the most active sample in BCB test was the ScEA, followed by Sc EtOH and ScB (55.82 ± 2.22, 53.11 ± 0.45 and 51.70 ± 1.97 %AA, respectively). While, there was a low activity for the apolar ScH fraction.

Moreover, correlations were calculated based on the average of the results by the Pearson test. This test calculates the linear correlation coefficient (r), a dimensionless number between –1 and 1, inclusive, reflecting the extent of a linear relationship between two datasets. The more the value of the coefficient comes close to the extremes, the more the correlation is present, in a positive or negative manner. The correlation value of 0 indicates no linear correlation. High correlation was found between the total polyphenol content and radical-scavenging activity (r_TPC/ABTS_ = 0.98; r_TPC/DPPH_ = 0.98; r_TPC/NO_ = 0.92; r_TPC/SO_ = 0.77) and reducing power (r_TPC/FRAP_ = 0.99) demonstrating as the polyphenols are the compounds mostly involved in the antioxidant activity [33]. The highest correlation was observed between the reducing power of the samples and the radical-scavenging activity by ABTS and DPPH methods (r_FRAP/ABTS_ = 0.99 and r_FRAP/DPPH_ = 1.00). Polyphenols are less involved in the inhibition of lipid peroxidation (r_TPC/BCB_ = 0.61). The antioxidant activity determined by the BCB method, a lipophilic system constituted by the emulsion *β*-carotene-linoleic acid, generally has a low correlation with the phenolic compounds and the other methods tested because of the effectiveness of lipophilic compounds [34]. Instead, terpenoids are clearly less involved in tested activities (Table 2).

**Figure 3:**
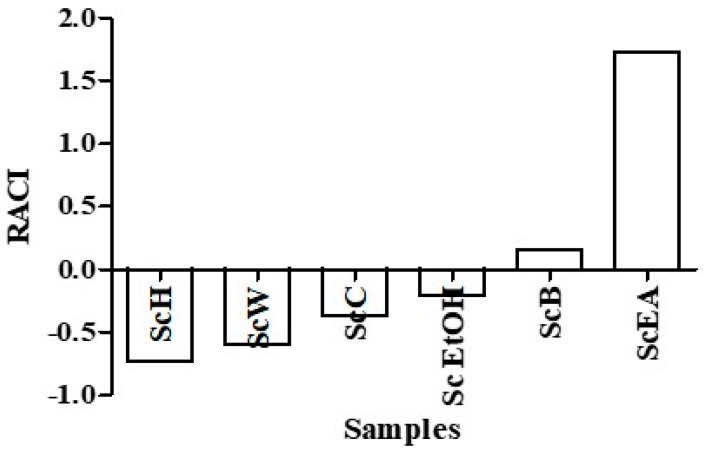
Relative Antioxidant Capacity Index (RACI) of *Senecio clivicolus* samples. Samples are crude ethanol extract (Sc EtOH), *n*-hexane fraction (ScH), chloroform fraction (ScC), ethyl acetate fraction (ScEA), *n*-butanol fraction (ScB), and water fraction (ScW).

The results of the antioxidant activity obtained by the above-described chemical methods have been integrated by calculating the Relative Antioxidant Capacity Index (RACI). The RACI index allows the comparison of phytocomplex antioxidant capacity derived from different chemical methods (ABTS, DPPH, SO, NO, FRAP, and BCB) even the TPC. It has recently been shown, that the results obtained with the method of Folin-Ciocalteu reagent can be interpreted to determine the total content of polyphenols, and as an alternative way to measure the total reducing capacity of the samples since the Folin reagent reacts with any reducing substance present in solution [22]. This index is dimensionless and measures the distance between the average of the results obtained and the raw data expressed in standard deviation units. RACI values obtained agrees with the results obtained so far (Figure 3) evidencing as ScEA fraction presented the highest value (1.73), followed by the ScB (0.16). The ScH fraction presented the lowest index (−0.73) and, therefore, a relative lack of antioxidant activity. In particular, the RACI values may be related to the high total polyphenol content of the ethyl acetate fraction.

To date, no study reported the antioxidant activity of *S. clivicolus* extracts, but Conforti et al. [28] evaluated the antioxidant activity of *Senecio gibbosus* aerial parts. This study reported as the methanol extract and ethyl acetate fraction had potent antioxidant property on DPPH radical as well as on lipid peroxidation. In particular, the paper reported an IC_50_ of 0.01 mg/mL for the ethyl acetate fraction of *S. gibbosus* on DPPH and showed higher inhibition than that ScEA (IC_50_ of 0.10 ± 0.00 mg/mL) obtained in present study. It is possible to explain these results with the different methods used to obtain the ethyl acetate fraction. In fact, Conforti et al. extracted the plant materials with methanol; then, the methanolic extract was acidified with 2.50% H_2_SO_4_ and partitioned with *n*-hexane, dichloromethane, and ethyl acetate. Moreover, in this paper the *n*-hexane fraction tested by DPPH assay was inactive, such as ScH. 

Similarly, the in vitro antioxidant activity of *Senecio inaequidens* and *Senecio vulgaris* by the DPPH assay showed that the ethyl acetate fraction gave the 61.60% and 44.57% of inhibition, respectively, at a concentration of 0.31 mg/mL [35]. In present study, ScEA showed a stronger inhibition compared to both species tested by Conforti et al. (90.55 ± 0.61% of inhibition at 0.31 mg/mL).

### 2.3. Identification and Quantification of Polyphenols by Mass Spectrometry

**Figure 4:**
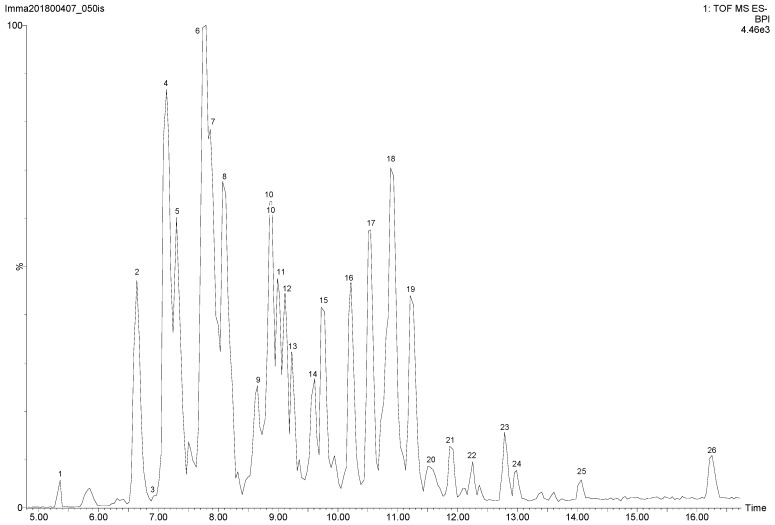
Ethyl acetate fraction *Senecio clivicolus* base peak intensity chromatogram (BPI).

The compounds previously identified in the aerial parts of *S. clivicolus* belong mainly to the class of alkaloids, eremophilanes and furanoeremophilanes [17,25]. To evaluate the compounds responsible of the measured antioxidant activity, ScEA fraction partitioned from ethanol extract of *S. clivicolus* aerial parts was subject to mass spectrometry analysis in negative ionization [36]. The LC-MS profile of ScEA is shown in Figure 4.

Several compounds (>26) were detected and tentative identification of most of them could be reached through accurate masses and fragmentation pattern and aided by the existing literature. As many as 19 of the compounds were of chlorogenic acid derivatives. Only eight phenolic compounds could be identified in the ethyl acetate fraction by comparing their retention times with those of the available commercial standards. In particular, these compounds were phenolic derivatives of benzoic acid (4-hydroxybenzoic acid (**1**)), phenylpropanoids, and in particular cinnamic acid derivatives (chlorogenic acid methyl ester (**3**), 3,5-di-*O*-caffeoyl quinic acid (**7**), 3,4-di-*O*-caffeoyl quinic acid (**8**), caffeic acid (**10**) and chlorogenic acid (**15**)), and flavonols (rutin (**4**) and quercetin-3-*O*-glucoside (**5**)). 

The most abundant was the 3,5-di-*O*-caffeoyl quinic acid (45.44 ± 0.91 mg/g DW), followed by 3,4-di-*O*-caffeoyl quinic acid (19.27 ± 0.68 mg/g DW). These phenolic compounds are known for their antioxidant properties [37,38,39,40,41,42]. 

As mentioned earlier, a number of the tentatively identified compounds were chlorogenic acid derivatives; in particular, five of the compounds with *m/z* 529.13 tentatively identified as feruloyl-caffeoylquinic acid isomers (**9**, **10**, **12**, **13**, and **20**). Upon fragmentation by CID, these compounds produced the ions at *m/z* 367, 353, 293, 193, 191, 179, 173, 161, 134, and 111 [43,44]. The signals at 9.23 (**13**) and 11.87 (**21**) min *m/z* 793.40 indicating the presence of an extra hexose sugar unit with respect to *m/z* 529.13 and were tentatively identified as chlorogenic acid methylester hexoside derivative; they produced ions at *m/z* 529, 191, 179, 173, and 161 [45]. The ESI-MS signals at *m/z* 601.22 (**15**, **16**), 617.23 (**15**, **16**), 779.23 (**17**), 763.23 (**18**, **19**), 819.26 (**22**), 807.30 (**20**, **23**), and 735.32 (**24**) were tentatively identified as chlorogenic acid derivatives; they produced the ions at *m/z* 353, 191, 179, 161 and 135 [46]. The ESI-MS signal at *m/z* 479.07 (**2**) was tentatively identified as quercetagetin-*O*-glucoside because it produced the ion *m/z* 317 of quercetagetin, and the typical ions *m/z* 166 and 139 [47]. The ESI-MS signals at 7.17 (**4**) and 9.61 (**14**) min *m/z* 493.09 were tentatively identified as mearnsetin-*O*-hexoside isomers and they gave dominant product ions *m/z* 478, 331 and 315 [48]. The ESI-MS signal at *m/z* 477.10 (**6**) was tentatively identified as isorhamnetin glycoside and it gave dominant product ions *m/z* 462, 315 and 299 [49]. Instead, the ESI-MS signal at *m/z* 519.10 (**11**) was tentatively identified as isorhamnetin-acetyl-glucoside because it produced the ion *m/z* 315 due to the loss of acetyl-glucose residue [50] (Table 3).

Previous reports evidenced as identified compounds are able to exert a strong antioxidant activity. In particular, Hung T.M. et al. (2006) reported 3,5-di-*O*-caffeoyl quinic acid and 3,4-di-*O*-caffeoyl quinic acid as potent scavengers of the neutral radical DPPH. These compounds demonstrated to be more potent than BHT used as a positive control [39]. 

Also, flavonoid derivative identified compounds in *S. clivicolus* EA fraction, showed to be effective when tested for their antioxidant activity. For example, rutin exhibited a strong DPPH radical scavenging activity and at 0.05 mg/mL showed 90.40% inhibition compared with BHT (58.80%) as reported from Yang et al. [40]. Moreover, rutin showed to be also effective in preventing lipid peroxidation. Razivi et al. [41] instead showed the cytotoxic, antimicrobial, and antioxidant activities of flavonol quercetin 3-*O*-glucoside.

The antioxidant activity analysis was effective in identifying the important polyphenolic contributors to the antioxidant capacity of *S. clivicolus* aerial parts, as assessed by TPC, TTeC, and in vitro antioxidant assays. The ethyl acetate fraction demonstrated the highest relative antioxidant capacity index and was subjected to mass spectrometry analysis. In the present study, the known antioxidant compounds such as phenolic derivatives of benzoic acid, cinnamic acid derivatives, and flavonoids were reported for the first time in *Senecio* genus. The most abundant of them were the known antioxidant 3,5-di-*O*-caffeoyl quinic acid and 3,4-di-*O*-caffeoyl quinic acid. 

## 3. Materials and Methods 

### 3.1. Chemicals, Reagents and Equipment

Solvents as *n*-butanol, chloroform, *n*-hexane, hydrochloric acid, ethanol, ethyl acetate, glacial acetic acid, methanol and phosphoric acid were purchased from Carlo Erba (Milan, Italy). 

Acetonitrile and formic acid were purchased from Merck (Wicklow, Ireland). Folin-Ciocalteu reagent 2N, sodium carbonate, 2,2′-azino-bis(3-ethylbenzothiazoline-6-sulfonic acid) (ABTS), potassium persulfate, 2,2-diphenyl-1-picrylhydrazyl (DPPH), potassium phosphate monobasic, *β*-nicotinamide adenine dinucleotide reduced form (NADH), phenazine methosulfate (PMS), nitrotetrazolium blue chloride (NBT), sodium nitroprusside dehydrate (SNP), sulfanilamide, *N*-(1-Naphthyl)ethylenediamine dihydrochloride, sodium acetate trihydrate, 2,4,6-tripyridyl-*s*-triazine (TPTZ), iron (III) chloride (FeCl_3_·6H_2_O), *β*-carotene, linoleic acid, and Tween 20 were purchased from Sigma-Aldrich (Milan, Italy). 

Standards as 6-hydroxy-2,5,7,8-tetramethylchroman-2-carboxylic acid (Trolox), ascorbic acid, butylhydroxytoluen (BHT), gallic acid, and Leucine-Enkephalin were purchased from Sigma-Aldrich (Milan-Italy) and Merck (Wicklow-Ireland), respectively. Standard as 3,4-di-*O*-caffeoyl quinic acid, 3,5-di-*O*-caffeoyl quinic acid, 4-hydroxybenzoic acid, caffeic acid, chlorogenic acid, chlorogenic acid methyl ester, quercetin-3-*O*-glucoside, and rutin were purchased from Extrasynthese (Genay, France). 

Water was deionized using a Milli-Q water purification system (Millipore, Bedford, MA, USA).

All spectrophotometric measurements were done in 96-well microplates or cuvettes on a UV–VIS spectrophotometer (SPECTROstar^Nano^ BMG Labtech, Ortenberg, Germany). 

LC-MS/MS analysis were performed on a Q-Tof Premier mass spectrometer (Waters Corporation, Milford, MA, USA) coupled to an Alliance 2695 HPLC system (Waters Corporation, Milford, MA, USA). Mass spectrometry quantification of the polyphenols was performed using multiple reaction monitoring (MRM) experiments using Waters Acquity (Waters Corporation, Milford, MA, USA) ultra-high performance liquid chromatography coupled with tandem mass spectrometry (UHPLC-MS/MS).

### 3.2. Plant Material and Sample Preparation

The aerial parts of *S. clivicolus* (Asteraceae family) were collected in the 2014, near the Aymaya population/community (18.45° S to 66.46° W; 3750 msnm), Bustillo province, Potosí department, Bolivia (Provincia di Nor Yungas, GPS coordinates −16.190040, −67.884402) dried at room temperature and crushed. A voucher specimen has been stored at the University of La Paz (number SC108-2014). Dried plant material (110.00 g) have been extracted by maceration in a darkness shaker set at 25 °C using 96% ethanol for 24 h at a solid to solvent ratio of 1:10 (*w*/*v*) per extraction (3 times). The extracts were filtered through a Buchner funnel (0.45 µm) and combined; subsequently supernantants have been dried using a rotary evaporator with a water bath set at 37 °C. 

Twenty grams of ethanol extract (Sc EtOH) were solubilized in water (400.00 mL) and subjected to liquid/liquid partitioning in triplicate using an equal volume of *n*-hexane, chloroform, ethyl acetate and *n*-butanol in order to separate the compounds on the basis of increasing solvent polarity. Then the *n*-hexane, chloroform, ethyl acetate, *n*-butanol, and water fractions (ScH, ScC, ScEA, ScB and ScW, respectively) were dried and stored in darkness at room temperature until further use. 

Sc EtOH and all the above-mentioned fractions were analyzed for their polyphenolic and terpenoids contents and their antioxidant activities.

### 3.3. Total Phenolic Content (TPC)

Total phenolic content (TPC) of *S. clivicolus samples* was determined by Folin-Ciocalteu assay as reported by Todaro et al. [52] with slight modification. The results were expressed as milligrams of gallic acid equivalents per gram of dried extract (mg GAE/g).

### 3.4. Total Terpenoid Content (TTeC)

The total terpenoid content (TTeC) was determined using linalool as standard reagent [53]. The absorbance was measured at 538 nm and the results were expressed as milligrams of linalool equivalents per gram of dried sample (mg LE/g).

### 3.5. Radical-Scavenging Activity

The radical scavenging capacity of *S. clivicolus* was evaluated by four different complementary in vitro assays. In particular, the synthetic coloured ABTS^+^ and DPPH^·^ radicals, and the biological super oxide anion (O_2_^−^) and nitric oxide (NO) radicals. The electron transfer involves reduction of a colored oxidant and the capacity of the samples to scavenge the radicals was monitored by spectrophotometer and quantified in Trolox equivalents, used as standard [15].

#### 3.5.1. ABTS Assay

The free radical-scavenging capacity of all *S. clivicolus* samples was studied using the 2,2′-azinobis-(3-ethylbenzothiazoline-6-sulfonic acid) diammonium salt (ABTS) radical assay, by the procedure described by Armentano et al. [54] with slight modification. The reaction between ABTS salt and potassium persulfate generates the ABTS^+^ radical after 16 h of incubation at room temperature. The reaction for scavenging the cationic radicals from the samples was monitored at 734 nm and the results were expressed as milligrams of Trolox equivalents per gram of dried extract (mg TE/g) by the Trolox standard curve.

#### 3.5.2. DPPH Assay

The radical-scavenging ability of samples was also evaluated by in vitro 2,2-diphenyl-1-picrylhydrazyl (DPPH) neutral radical and, also in this case, Trolox was used as standard. The reaction was monitored at 515 nm and the data were expressed as milligrams of Trolox equivalents per gram of dried extract (mg TE/g) [22].

#### 3.5.3. Super Oxide Anion Scavenging Activity (SO)

Superoxide anion radicals (O_2_^−^) were generated in vitro by the NADH/PMS system as described by Russo et al. [55]. The scavenging activity of samples on the inhibition of formazan formation was monitored at 560 nm by spectrophotometer and ascorbic acid was used as positive control. The results were expressed as the concentration inhibiting 25% of radical inhibition in mg/mL (IC_25_).

#### 3.5.4. Nitric Oxide Radical Scavenging Activity (NO)

The nitric oxide radical (NO) was spontaneously generated at physiological pH from nitroprusside. Then, the nitric oxide interacts with oxygen to give nitrite ions that can be determined spectrophotometrically by Griess reagent [55]. Results were expressed as IC_25_ of radical inhibition in mg/mL and ascorbic acid was used as positive control.

### 3.6. Ferric Reducing Antioxidant Power Assay (FRAP)

FRAP reagent was prepared fresh before the experiment by mixing 300 mM sodium acetate buffer at pH 3.60, 20 mM FeCl_3_·6H_2_O in distilled water and 10 mM TPTZ in 40 mM HCl in a ratio of 10:1:1. The reaction was monitored at 593 nm and Trolox was used as reference antioxidant standard. FRAP values were expressed as milligrams of Trolox equivalents per gram of dried extract (mg TE/g) [56]. 

### 3.7. β-Carotene Bleaching Assay (BCB)

Lipid peroxidation inhibition was evaluated by the *β*-carotene bleaching method (BCB) [57]. The *β*-Carotene emulsion (950.00 µL) was mixed with 50.00 µL of sample at 1.00 mg/mL and BHT was used as positive control, then the mixture (250.00 µL) was transferred into a 96-well plate. Outer wells were filled with 250.00 μL of water to provide a large thermal mass because the reaction was temperature-sensitive and close temperature control throughout the plate was essential in this assay [58]. The microplate was placed at 50 °C for 3 h and the absorbance was measured at 470 nm at 0′, 30′, 60′, 90′, 120′, 150′, and 180′. The results were expressed as percentage of *β*-carotene bleaching inhibition (% Antioxidant Activity, %AA).

### 3.8. Identification and Quantification by Liquid Chromatography Mass Spectrometry

LC-MS characterization was carried out on a Q-Tof Premier mass spectrometer (Waters Corporation, Milford, MA, USA) coupled to an Alliance 2695 HPLC system (Waters Corporation, Milford, MA, USA) as described previously [59,60]. Accurate mass measurements of the analytes were achieved through the use of an internal reference compound (leucine-enkephalin). The separation of the compounds was performed on an Atlantis T3 C18 column (Waters Corporation, Milford, USA, 100.00 mm × 2.10 mm; 3.00 µm particle size) maintained at 40 °C and using 0.10% aqueous formic acid (solvent A) and 0.10% formic acid in acetonitrile (solvent B). A stepwise gradient from 10% to 90% solvent B was applied at a flow rate of 0.30 mL/min for 25 min. Electrospray mass spectra data were acquired on a negative ionization mode for a mass range *m/z* 100 to *m/z* 1000. Cone voltage and capillary voltage were set at 30 V and 3 kV, respectively. Collision induced fragmentation (CID) of the analytes was achieved using 12 eV to 30 eV energy with argon as the collision gas.

For the quantification of the polyphenols in the ethyl acetate fraction of *S. clivicolus*, Waters Acquity (Waters Corporation, Milford, MA, USA) ultra-high performance liquid chromatography coupled with tandem mass spectrometry (UHPLC-MS/MS) was used. The compounds were separated on a Waters Acquity HSS T3 C18 column (2.10 × 100.00 mm; 1.80 µm particle size) using a binary solvent system consisting of water containing 0.10% formic acid (mobile phase A) and acetonitrile containing 0.10% formic acid (mobile phase B). The following gradient program was carried out: 0–2.50 min 2% B, 2.50–3.00 min 10% B, 3.00–7.50 min 15% B, 7.50–8.50 min 35% B, 8.50–9.50 min 98% B and 9.50–10.00 min 2% B at a flow rate of 0.50 mL/min. The injection volume for all the samples and the standards was 3.00 μL. All the standards in the concentration ranging from 0.01 to 50.00 μg/mL were dissolved in 80% methanol. Multiple reaction monitoring (MRM) quantitative method was developed for each of the standard compound using the Waters Intellistart software, and the quantifications of the data were carried out using the Waters TargetLynx™ software. The ionization source conditions were as follows: capillary voltage 3 kV, cone voltage 35 V, source temperature 150 °C, desolvation temperature 350 °C, desolvation gas flow 200 L/h, cone gas flow 50 L/h, and collision gas flow 0.10 mL/min. The column temperature was maintained at 40 °C.

### 3.9. Statistical Analysis

All data were expressed as mean ± standard deviation (SD) of three independent experiments performed in triplicate. To verify the correlation among used methods, the *p* value of 0.05 or less was considered significant and calculated by one-way analysis of variance (ANOVA). Pearson coefficient and all other statistical calculations were determined using GraphPad Prism 5 Software (San Diego, CA, USA).

## 4. Conclusions

In this report the ethanol extract of *S. clivicolus* aerial parts was subjected to liquid/liquid fractionation using solvent with increasing polarity. The antioxidant activity, determined by six complementary in vitro assays, showed different values among fractions. Relative Antioxidant Capacity Index (RACI), evidenced *S. clivicolus* ethyl acetate fraction as the most active. More in detail, fraction reported activity was: *n*-hexane < water < chloroform < *n*-butanol < ethyl acetate. Moreover, this study reports the profile of the phenolic compounds in *S. clivicolus* ethyl acetate fraction. The phytochemical investigation allowed the identification or tentative identification of 30 compounds and confirms that the LC-MS-based profiling is a powerful technique for the phenolic characterization. This first report on *S. clivicolus* phenolics demonstrated as it could be considered a rich source of health promoting compounds, particularly chlorogenic acid, and flavonoid derivatives. Hung et al. (2006) demonstrated as this compounds are able to exert a protective role against oxidative stress. In fact chlorogenic acid, the most abundant isomer among caffeoylquinic acid isomers, is a biologically active polyphenol, which play several biological activities such as antioxidant [42]. 

In conclusion, based on the observed results in conjunction with the existing literature, it is anticipated that *S. clivicolus,* especially its polar extracts, might be used for mitigating human and animal diseases. The presence of potential nutraceuticals is suitable and promising for the development of safe food products, natural additives and cosmetics. Its compounds, previously tested for their biological activity, seems to explain the most of the measured antioxidant activity. This will further trigger extensive research for better understanding of the impact of *S. clivicolus* phenolic compounds on health.

## Figures and Tables

**Table 1:** Results of ABTS, DPPH and super oxide (SO) scavenging activity, ferric reducing antioxidant power (FRAP) and *β*-carotene bleaching (BCB) of *S. clivicolus* samples.

Samples	ABTS (mgTE/g)	DPPH (mgTE/g)	SO (IC_25_ mg/mL)	FRAP (mgTE/g)	BCB %AA
**Sc EtOH**	137.87 ± 1.45 ^d^	63.42 ± 0.78 ^b^	0.37 ± 0.02 ^d^	93.08 ± 1.12 ^d^	53.11 ± 0.45 ^d,e^
**ScH**	28.94 ± 2.50 ^a^	nc	0.16 ± 0.01 ^b,c^	12.98 ± 1.04 ^a^	4.75 ± 0.23 ^a^
**ScC**	55.93 ± 2.24 ^b^	12.70 ± 0.94 ^a^	0.14 ± 0.01 ^b^	23.90 ± 1.32 ^b^	44.58 ± 0.96 ^c^
**ScEA**	409.53 ± 9.53 ^f^	317.53 ± 5.81 ^d^	0.08 ± 0.00 ^a^	507.66 ± 5.26 ^f^	55.82 ± 2.22 ^e^
**ScB**	208.37 ± 3.21 ^e^	119.54 ± 6.71 ^c^	0.20 ± 0.01 ^c^	184.18 ± 4.59 ^e^	51.70 ± 1.97 ^d^
**ScW**	107.01 ± 0.94 ^c^	55.58 ± 1.07 ^b^	0.64 ± 0.04 ^e^	53.41 ± 2.55 ^c^	23.28 ± 0.70 ^b^

Samples are crude ethanol extract (Sc EtOH), *n*-hexane fraction (ScH), chloroform fraction (ScC), ethyl acetate fraction (ScEA), *n*-butanol fraction (ScB) and water fraction (ScW). Data are expressed as means ± standard deviation from three experiments; mg GAE/g = mg of gallic acid equivalents per gram of dried sample; mg TE/g = mg of Trolox equivalents per gram of dried sample; IC_25_ mg/mL = concentration of the sample required to inhibit the activity of the radical by 25%; %AA = percentage of antioxidant activity at initial sample concentration of 1 mg/mL; different superscripts in the same row indicate significant difference (*p* < 0.05); nc = not calculable.

**Table 2:** Pearson correlation coefficients calculated among tested *Senecio clivicolus* extract and fractions.

	TPC	TTeC	ABTS	DPPH	SO	NO	FRAP	BCB
**TPC**	1.00							
**TTeC**	−0.69	1.00						
**ABTS**	0.98	−0.64	1.00					
**DPPH**	0.98	−0.68	0.99	1.00				
**SO**	0.77	−0.56	0.65	0.70	1.00			
**NO**	0.92	−0.75	0.89	0.93	0.84	1.00		
**FRAP**	0.99	−0.70	0.99	1.00	0.75	0.94	1.00	
**BCB**	0.61	0.01	0.65	0.59	0.29	0.41	0.58	1.00

Total phenolic content (TPC); total terpenoids content (TTeC); ABTS assay; DPPH assay; Super oxide anion scavenging activity (SO); nitric oxide radical scavenging activity (NO); Ferric reducing antioxidant power assay (FRAP); *β*-carotene bleaching assay (BCB).

**Table 3:** Liquid chromatography-tandem mass spectrometry (LC-MS/MS) of ethyl acetate fraction of *Senecio clivicolus*. Identification of compounds based on *m/z*, and fragmentation pattern and retention time of standards. Quantities of the detected phenolic compounds were determined using commercial standards.

Peak No.	RT (min)	ESI (−) MS Observed	ESI (−) MS Calc.	Molecular Formula	MS/MS	Tentative Identity	mg/g DW	Reference
1	5.39	137.0242	137.0239	C_7_H_5_O_3_	93	4-Hydroxybenzoic acid	2.25 ± 1.31	[36]
2	6.64	479.0779	479.0826	C_21_H_19_O_13_	317, 166, 139	Quercetagetin-*O*-glucoside	nq	[47]
3	6.92	367.1029	367.1029	C_17_H_19_O_9_	161, 85	Chlorogenic acid methylester	2.57 ± 0.07	[36]
4	7.17	493.0982	493.0982	C_22_H_21_O_13_	478, 331, 315, 287, 271, 244, 166	Mearnsetin-*O*-glucoside isomer	nq	[48]
		609.1461	609.1456	C_27_H_29_O_16_	300, 285, 271, 255, 179, 151	Rutin	0.16 ± 0.02	[36]
5	7.25	463.0894	463.0877	C_21_H_19_O_12_	300, 271, 255, 179, 151	Quercetin-3-*O*-glucoside	0.84 ± 0.05	[36,51]
6	7.80	477.1067	477.1033	C_22_H_21_O_12_	462, 315, 299, 271, 254, 243, 227, 151	Isorhamnetin glycoside	nq	[49]
7	7.91	515.1174	515.1190	C_25_H_23_O_12_	179, 135	3,5-di-*O*-caffeoyl quinic acid	45.44 ± 0.91	[36]
8	8.13	515.1169	515.1190	C_25_H_23_O_12_	179, 135	3,4-di-*O*-caffeoyl quinic acid	19.27 ± 0.68	[36]
9	8.66	529.1370	529.1346	C_26_H_25_O_12_	367, 349, 191, 179, 173, 161, 135, 133, 101, 93	Feruloyl-caffeoylquinic acid isomer	nq	[43,44]
10	8.86	179.0353	179.0344	C_9_H_7_O_4_	135, 79	Caffeic acid	1.88 ± 0.13	[36]
		529.1370	529.1346	C_26_H_25_O_12_	367, 349, 191, 179, 173, 161, 135, 101	Feruloyl-caffeoylquinic acid isomer	nq	[43]
11	8.99	519.1039	519.1139	C_24_H_23_O_13_	504, 315, 299, 285, 271, 243, 191	Isorhamnetin-acetyl-glucoside	nq	[50]
12	9.12	529.1370	529.1346	C_26_H_25_O_12_	367, 349, 191, 179, 173, 161, 135, 101	Feruloyl-caffeoylquinic acid isomer	nq	[43]
13	9.23	529.1370	529.1346	C_26_H_25_O_12_	367, 349, 191, 179, 173, 161, 135, 101	Feruloyl-caffeoylquinic acid isomer	nq	[43]
		793.4029	793.4010	C_41_H_61_O_15_	529, 397, 353, 219, 191, 179, 173, 161, 101, 71	Chlorogenic acid methylester hexoside derivative	nq	[45]
14	9.61	493.0982	493.0982	C_22_H_21_O_13_	331, 316, 179, 161, 135, 133, 101	Mearnsetin-*O*-glucoside isomer	nq	[48]
15/16	9.72/10.21	353.0885	353.0873	C_16_H_17_O_9_	191, 179, 173, 93, 85	Chlorogenic acid	2.33 ± 0.45	[36]
		617.2367	617.2387	C_35_H_37_O_10_	353, 245, 191, 179, 173, 161, 135	Chlorogenic acid derivative	nq	[46]
		601.2267	601.2285	C_31_H_37_O_12_	439, 353, 263, 191, 179, 173, 161, 135, 85	Dicaffeoyl-methoxyoxaloylquinic acids	nq	[46]
17	10.55	779.2360	779.2340	C_43_H_39_O_14_	515, 375, 353, 335, 191, 179, 173, 161, 155, 135, 111, 93	Chlorogenic acid derivative	nq	[46]
18/19	10.88/11.21	763.2333	763.2332	C_50_H_35_O_8_	515, 353, 191, 179, 173, 161, 135, 110	Dicaffeoylquinic acid derivative	nq	[46]
20	11.50	529.1370	529.1346	C_26_H_25_O_12_	367, 353, 293, 193, 191, 179, 173, 161, 134, 111	Feruloyl-caffeoylquinic acid isomer	nq	[43]
		807.3002	807.3017	C_46_H_47_O_13_	353, 335, 191, 179, 173, 161, 155, 135	Chlorogenic acid derivative	nq	[46]
21	11.87	793.2888	793.2860	C_45_H_45_O_13_	353, 191, 179, 173, 161, 155, 135	Chlorogenic acid derivative	nq	[45]
22	12.25	819.2629	819.2618	C_28_H_51_O_27_	353, 335, 191, 179, 173, 161, 155, 135	Chlorogenic acid derivative	nq	[46]
23	12.79	807.3002	807.3017	C_46_H_47_O_13_	353, 335, 191, 179, 173, 161, 155, 135	Chlorogenic acid derivative	nq	[46]
24	12.99	735.3240	735.3228	C_37_H_51_O_15_	353, 335, 191, 179, 173, 161, 135	Chlorogenic acid derivative	nq	[46]
25	14.70	675.3726	675.3744	C_37_H_55_O_11_	415, 397, 277, 235, 161, 143, 125, 119, 113, 101, 89	unknown	nq	
26	16.25	480.3110	480.3087	C_27_H_44_O_7_	255, 242, 224, 168, 153, 79	unknown	nq	

nq: not quantified.
